# Does cerebrospinal fluid multiplex polymerase chain reaction meningitis panel help manage children with meningitis?

**DOI:** 10.21203/rs.3.rs-7871089/v1

**Published:** 2025-12-17

**Authors:** Supatida Tengsupakul, Madhuri Mulekar, Bibek Raj Bista, Paul Maertens, Kamal Sharma

**Affiliations:** University of South Alabama; University of South Alabama; University of Texas Health Science Center; University of South Alabama; University of South Alabama

**Keywords:** Pediatrics, lumbar puncture, pretreatment, viral meningitis

## Abstract

**Back round:**

Meningitis has significant clinical morbidity and mortality in children. Mortality in children, especially neonates, is high due to diagnostic difficulty. Diagnosis of pediatric meningitis is traditionally made using CSF parameters and cultures. The performance of cerebrospinal fluid (CSF) multiplex polymerase chain reaction (PCR) meningitis panel needs further evaluation in the pediatric population.

**Methods:**

Medical records of children with a positive CSF multiplex panel (June 1, 2016 to August 31, 2018) were reviewed retrospectively. We extracted data about antimicrobials used, laboratory and culture results, duration of antibiotic treatment and hospital stay.

**Results:**

A total of 79 children (age, 1 d to 12 y) were identified, including 58 children with viral (73%) and 21 children (27%) with bacterial meningitis. The most frequent viruses were enterovirus in all age groups. Empiric antibiotics were discontinued within 24 h in 14 of 40 patients (35%) positive for viral meningitis. Specific antiviral therapy was rapidly initiated in 6 out of 58 patients (10%). Antibiotics were stopped or never initiated when viral etiology was identified. Even when the CSF sample was inadequate for analysis, the pathogenic organism was identified particularly helpful in the infants. In antibiotic pretreated patients, the organism was identified even when the cultures were negative. CSF multiplex panel identified 13 patients who had bacterial meningitis despite negative CSF cultures. Twelve of these 13 patients (92%) were pretreated with antibiotics. Modification of antibiotic therapy was never the result of CSF multiplex panel. Duration of antibiotic treatment and hospital stay were substantially shorter in patients with viral as opposed to bacterial meningitis.

**Conclusions:**

CSF multiplex panel saves brain as it is rapid, specific, and highly sensitive. It also reduces the healthcare cost, and its use is highly recommended in a pediatric population.

## Background

In children with or without disseminated illness, meningitis has significant clinical morbidity and mortality [1]. Mortality is multifactorial and can vary with the causative pathogen. Despite the availability of effective antibiotics to treat bacterial meningitis, 50% of children may experience neurologic sequelae, and mortality is high (5% to 30%), especially in neonates, in part because of diagnostic difficulty [2, 3].

In Pediatrics, the incidence of meningitis is highest under 6 months of age [4]. Clinical examination is not sufficient for diagnosis as symptoms and signs of meningitis in children are variable and nonspecific [5]. In neonates, clinical presentation varies even more. The most common symptoms prompting diagnostic lumbar puncture in the emergency room include fever, seizures, and/or unexplained altered mental status [3]. Laboratory confirmation of bacterial meningitis is ascertained by cerebrospinal fluid (CSF) Gram stain and culture. Treatment with empiric antibiotics before obtaining CSF studies can affect the sensitivity of culture and Gram stain [6]. The sensitivity of CSF Gram stain is 60% to 80% in patients without antibiotic pretreatment and decreases by 20% with antibiotic pretreatment [7]. CSF bacterial culture is positive in 8% of patients who have meningitis [8] but is a useful diagnostic test even though results may not be available for two days, because it provides information about the pathogen and antimicrobial sensitivity [9]. Parameters such as CSF protein level, glucose level, and white blood cell (WBC) count may be inconsistent in differentiating viral from bacterial infections and nonspecific to provide treatment direction [7].

The purpose of the present study is to evaluate utility of positive CSF PCR results.

## Materials & Methods

### Study design

In this single-center retrospective study, we included all children under 18 years of age who had a positive CSF multiplex PCR panel (FilmArray Meningitis/Encephalitis (ME) Panel, BioFire Diagnostics, Salt Lake City, UT) and were admitted from June 1, 2016 to August 31, 2018. In the case of multiple positive CSF specimens for the same patient, only the first result was included in the study to avoid double entry of the same patient which would skew the data. Patients were grouped by age (< 30 d; 30 to 90 d; >90 d) into three categories. Clinical data was extracted from the electronic medical records of patients after institutional board approval. The results of the CSF multiplex panel were deidentified. Laboratory data included serum WBC count, C-reactive protein (CRP), CSF WBC count, protein level, and glucose level. Antibiotic pretreatment was defined as antibiotic treatment received within seven days before lumbar puncture. Respiratory distress was defined as tachypnea for age, retractions, or grunting as defined in the Pediatric Advanced Life Support manual [10].

The CSF data was categorized into normal and abnormal groups as follows. Normal CSF WBC was defined as < 20 cells/mm^3^ for age ≤ 28 d, < 10 cells/mm^3^ for age 29–60 d and < 5 cells/mm^3^ for age > 60 d [11]. Normal CSF protein level was defined as < 130 mg/dL for age ≤ 28 d, < 100 mg/dL for age 29–60 d, or < 70 mg/dL for age > 60 d. Normal CSF glucose level was defined as 37 to 75 mg/dL for all ages [11]. Traumatic lumbar puncture was defined by CSF RBC count > 10,000 cells/mm^3^. CSF cell count and protein were adjusted according to the correction formula [12, 13].

### Statistical analysis

Data was entered into a spreadsheet (Excel, Microsoft, Redmond, WA) and analyzed with statistical software (JMP Pro v 14.2.0–16.2.0 and 18.0.2, SAS Inc., Cary, NC). Numerical variables such as age and duration of hospital stay were summarized using mean and range. Categorical variables such as pathogen and gender were summarized using count and percent. Mean outcomes for numerical variables were compared for bacterial/viral meningitis patients using a 2-sample t-test and that for categorical variables using Fisher’s or Chi-square test. Odds ratios and their 95% confidence intervals (CI) were computed to measure the association between bacterial/viral infection and factors such as normal CSF protein, glucose, WBC counts. Comparisons within the age groups were not performed because of small sample sizes. Statistical significance was defined as *P* < 0.05.

## Results

There were 79 patients aged 1 d to 12 y (0.7 ± 2 y) who had multiplex panels showing at least one pathogen. No samples showed multiple pathogen detections. Most patients (73%) had viral meningitis, and the mean age did not differ significantly between patients with viral versus bacterial meningitis. Fever was the presenting symptom in most patients (65%), and 4 neonates with viral meningitis had hypothermia. Respiratory distress was less frequent in patients with viral than with bacterial (7% vs 48%) meningitis (Table 1). Comparison of serum WBC and CRP levels for patients with bacterial/viral meningitis showed that mean CRP level was significantly higher among those with bacterial infection than viral (4.41 vs 3.29, 2-sample t-test, *p* < 0.0001). Comparison of odds of viral vs bacterial meningitis using odds ratio showed that with each unit elevation of CRP level, the odds of bacterial meningitis will increase by a factor of 1.28 compared to that of viral meningitis (OR = 1.28, *P* = 0.002); however, no such association was observed with serum WBC (OR = 1.02, *P* = 0.73) with fairly similar odds for both types of infections (Table 2). The most frequently observed viral pathogen was enterovirus in patients from all age groups, and the most frequently observed bacterial pathogens were *Streptococcus agalactiae* for patients < 30 d old, *Escherichia coli* for patients 30–90 d old, and *Haemophilus influenzae* for patients > 90 d old (Table 3). Some pathogens in the multiplex panel were not identified in any patient, such as *Listeria monocytogenes*, *Neisseria meningitidis*, *Cryptococcus neoformans*, and *Cryptococcus gattii*.

CSF multiplex identified 37 (47%) children with enterovirus, 11 (14%) with human herpes virus 6 (HHV-6) and 4 (5%) with parechovirus meningitis (Table 4). Antibiotic therapy was never initiated in 8 children with enterovirus meningitis (22%), 3 children with HHV-6 meningitis (27%), and 1 with parechovirus meningitis (25%). In 14 of 40 patients (35%) with viral meningitis who had received empiric antibiotic treatment, the antibiotics were discontinued within 24 h after CSF sampling.

The study of glucose, protein, and cell count was not performed in 4 patients due to the small volume of CSF available for analysis. One infant was a 12-month-old boy who presented with seizures. CSF multiplex panel was positive for HHV-6. Three remaining infants were less than 30 days old. The CSF multiplex panel was positive for viral meningitis in all 3 infants. Infant 1 was a two-day-old newborn male with respiratory distress who received empiric antibiotics for 24 hours and underwent a spinal tap due to progressive elevation of CRP (8.4 mg/dL) on the second day of life. CSF multiplex panels were positive for cytomegalovirus (CMV). CMV culture was positive in CSF and urine. The infant received oral valganciclovir for 6 months and had a normal outcome at 6 years of age. Infant 2 is a 17-day-old male who presented with decreased oral intake, fussiness, and subjective fever. Initial spinal tap was unsuccessful, and he was given empirical antibiotics however, acyclovir was not started. After 2 days, due to onset of seizures, spinal tap was repeated yielding 1 ml of CSF. CSF multiplex panel was positive for herpes simplex virus 2 (HSV-2) and antibiotic therapy was discontinued when CSF bacterial culture was negative. He was treated with acyclovir for 6 months. He required a ventriculoperitoneal shunt and only suffers from a mild global developmental delay. At the age of 8, he functions like a 6-year-old child. Infant 3 is a 30 day-old-female who presented with seizure and had a CSF multiplex panel positive for HHV-6. Patient improved without therapy.

Data about CSF WBC, protein, and glucose was available for 75 patients when patients from all three age groups were combined into one. As expected, normal CSF protein (80% vs 33%, Chi sq test, *p* < 0.0001) and glucose (89% vs 57%, Chi sq test, *p* = 0.01) levels were noted more frequently in children with viral meningitis than with bacterial meningitis. CSF cell count, however, was not significantly different (*P* = 0.46) in patients with viral or bacterial meningitis. Comparisons within three different age groups were not performed due to small sample sizes (Table 3). The odds of normal CSF protein and glucose were respectively 7.71 (*p* < 0.0001) and 6 (*p* = 0.01) times higher among patients with viral meningitis than with bacterial meningitis.

Among 54 infants with positive viral CSF multiplex panel, 44 (81%) were less than 90 d old, 38 (86%) had normal CSF glucose and 36 (82%) had normal CSF protein. Among these infants, one infant of interest was a 42-day-old male presenting with worsening diffuse papular rash which became vesicular and pustular. He had cold symptoms and fever and underwent a septic workup. CSF was normal except for CSF multiplex panel which was positive for varicella zoster virus (VZV). The infant was treated with acyclovir. In two infants less than 90 days old, CSF protein was elevated while CSF glucose and WBC were normal. One infant was extremely premature, 24 + 5/7 weeks small for a gestational age female, born to a G1POOOO mother with sickle cell disease. At 48 days of life, recurrent episodes of apnea/ bradycardia were evaluated with spinal tap. CSF WBC count was 3 cells/mm^3^, red blood was 510 cells/mm^3^ and CSF protein 169 mg/dL. CSF multiplex panel showed HSV-1. Patient was treated with intravenous acyclovir for 21 days and oral acyclovir for 6 months. At a 2 year and 4 months follow-up, the patient was noted to have short stature and mild language developmental delay. Low CSF glucose was seen in 6 infants with viral meningitis in infants less than 90 days old. Among those 6 less than 90 d old infants, 4 had all abnormal CSF parameters including protein and WBC. All these infants had CSF multiplex panel positive for enterovirus meningitis and negative HSV workup. Infant 1, a 9-day-old male newborn, received acyclovir for two days until full HSV workup was negative and empiric antibiotics for three days until bacterial culture was negative. Infants 2 and 3 were a set of female twins who presented with hypothermia at the age of 17 days, and Infant 4 was a 39-day-old male who developed fever. The acyclovir was not given to these three infants.

In all 10 over 90 d old children with viral meningitis, normal CSF glucose was reported (Table 3). In 7 of 10 (70%) patients CSF protein was normal and elevated in remaining 3 patients. One of them is an 8-year-old male with fever, behavioral changes and status epilepticus and required intubation and mechanical ventilation. His CSF protein and glucose were normal while CSF WBC (23 cells/mm^3^) and RBC (50 cells/mm^3^) were elevated. He was found to have HSV-1 meningitis by CSF multiplex panel and as a result he was given intravenous acyclovir and empiric antibiotics for meningitis. Antibiotics was not stopped as he was pretreated before the spinal tap. He was intubated for status epilepticus and acute respiratory failure and successfully extubated after 13 days of mechanical ventilation. He was treated with intravenous acyclovir for 21 days and oral acyclovir for 5 months. Patient was discharged after 27 days of hospitalization to follow up with occupational therapy.

Twenty-one patients had bacterial meningitis identified by the multiplex panel, and CSF culture results were available for all of them. In 8 (38%) of them, CSF culture results confirmed the pathogen identified by the multiplex panel. CSF cultures showed no growth in remaining 13 (62%) patients who had bacterial pathogens identified by CSF multiplex panel (Table 5). A negative CSF culture was the result of antibiotic pretreatment in the majority (92%) of these 13 patients. In two patients, blood culture results confirmed the pathogen identified by the CSF multiplex panel (Table 6). All other patients had negative blood cultures. Furthermore, in these 13 patients, a wide range of serum and CSF biomarker results were observed, and most of them had blood and urine cultures either not available or showing no growth (Table 6).

When the antibiotic duration and hospital stay were analyzed (Table 5), as expected, patients with bacterial meningitis had significantly longer treatment durations and longer hospital stays. Mean duration of antibiotic treatment was 2 d (range, 0–6 d) in children with viral meningitis and 18 d (range, 9–28 d) in children with bacterial meningitis (*P* = 0.0015). Mean duration of hospital stay was 4 d (range 1–23 d) in children with viral meningitis and 22 d (range 16–27 d) in children with bacterial meningitis (*P* < 0.0001).

## Discussion

Real-time polymerase chain reaction (PCR) amplifying a single target pathogen has been in use in commercial laboratories in the United States for detecting pathogens since the 1990s [14]. In 2015, the U.S. Food and Drug Administration (FDA) approved the first multiplex panel for detection of CNS infections [15]. The CSF multiplex panel detects 14 common pathogens, including six bacteria, seven viruses, and one fungus. The pathogens included in CSF multiplex are CMV, enterovirus, HSV-1 and 2, HHV-6, human parechovirus, VZV, *Escherichia coli K1, Haemophilus influenzae, Listeria monocytogenes, Neisseria meningitidis, Streptococcus agalactiae, Streptococcus pneumoniae*, and Cryptococcus neoformans/Cryptococcus gattii. Multiplex panel decreases the need for single pathogen testing, allowing labs to provide more comprehensive results with less resources. Studies about the efficacy of this CSF multiplex panel have shown high sensitivity (86%−100%), specificity (92.8%−100%), positive predictive value (85%), and negative predictive value (98%) [16].

CSF parameters can sometimes distinguish viral and bacterial meningitis. In our study, normal CSF protein and glucose go in favor of a viral etiology. However, CSF pleocytosis, a sensitive marker for inflammation, is less predictive (Table 3). CSF parameters are a poor predictor of the organism causing meningitis and normal CSF parameters could be misleading. Some patients in our study suffered from serious, treatable meningitis and would have been missed if CSF multiplex had not been performed. In our study, 4 infants with positive bacterial CSF multiplex had all normal CSF parameters. All of them were neonates. In neonates, bacterial meningitis confirmed with positive cultures is possible with normal CSF parameters including CSF WBC count [17].

The present study confirmed that CSF multiplex panel was useful in the early diagnosis and treatment of pediatric meningitis. First, CSF multiplex panel is rapid compared to slow conventional culture methods. FilmArray CSF multiplex panel analyzes multiple target sequences simultaneously, enabling the testing for multiple pathogens in a single test with a turnaround time of about 1 hour [18, 19]. By quickly identifying the cause of meningitis, clinicians can make more informed decisions. In our study, multiplex panel enabled rapid identification of a treatable viral meningitis in 6 out of 58 children with viral meningitis (10%). HSV was identified by CSF multiplex in 4 children, two with HSV-1 and two with HSV-2. Two HSV infants were less than 90 days old. First one is a 19-day-old male infant with no CSF parameter available. Treatment was initiated without delay. Second one is 49-day-old female infant who had normal CSF glucose and cell count but high CSF protein. The third infant is an 8-month-old male. CSF WBC and CSF glucose were normal but CSF protein was high. Fourth HSV patient is an 8-year-old female who had CSF WBC abnormal but had normal CSF protein and glucose. Fifth patient is a 2-day-old male for whom CSF multiplex panel identified CMV meningitis but CSF sample was not sufficient for analysis. Sixth patient is a 42-day-old male with VZV identified by CSF multiplex panel who had all CSF parameters normal. The above-mentioned patients would have been missed had there been no CSF multiplex panel available showing the vital importance of the CSF multiplex panel. Specific antiviral therapy was initiated without delay. In addition, CSF multiplex panel identified non-treatable pathogens, such as enterovirus, HHV-6, and parechovirus in 52 out of 58 patients with viral meningitis (90%). When non-treatable viral pathogens were identified by CSF multiplex panel, acyclovir, which is nephrotoxic and vesicant, was discontinued. In addition, discovery of non-treatable viral pathogens prevented unnecessary antimicrobial use. In our study, all non-treatable viral pathogens detected in CSF multiplex panel were never associated with other CSF pathogens. Antibiotics were never started in 25% of children with non-treatable viral pathogens and were only given for 24 hours or less in 35%. Because bacterial meningitis is a life-threatening medical emergency, patients have historically and currently been started on broad-spectrum antimicrobial therapy as a precaution while awaiting CSF parameters and cultures. Unnecessary antibiotic use was increasing the risk of developing multidrug-resistant organisms and leading to serious side effects and complications, including *C. difficile* infection, allergic reactions, and organ toxicity [20]. CSF multiplex panel identified bacterial meningitis in 27% of patients. In our study, multiplex panel did not help in simplifying antibiotic regimen in bacterial meningitis and in each case antibiotic therapy was only modified after obtaining culture and sensitivity results. Continuing the use of Gram stain and bacterial cultures is still a valid recommendation to confirm the results of the CSF multiplex panel [21] and guide antimicrobial therapy based on susceptibility results. Contrary to a previous report [22], we found that the multiplex panel does not necessarily simplify antibiotic management in bacterial meningitis. Currently CSF multiplex panel does not provide information about antibiotic susceptibility [9]. None of our patients had meningococcal meningitis. Rapid identification of such pathogens would not only improve patient outcomes but also would allow the implementation of necessary public health measures like antibiotic prophylaxis needed within 24 hours to prevent outbreaks [23].

Second, CSF multiplex panel is highly sensitive. The multiplex panel can identify bacterial pathogens that are missed by Gram stain and culture [24]. The sensitivity of culture and Gram stain is variable with type of organisms and demographics, but was reported to be 8 percent and 60 percent, respectively [8]. Sample volume can be small and still provide quick identification of CSF pathogens in an infected patient. In our study, the four patients with inadequate CSF samples, too small for full analysis, were less than 12 months of age. Two infants, one infant with HSV meningitis and one with congenital CMV, were treated without knowledge of CSF parameters. These cases highlight the importance of CSF multiplex panel as diagnosis would have been delayed or missed. Furthermore, CSF multiplex panel can detect a pathogen’s genetic material (DNA or RNA) even if the organism has been killed or suppressed by antibiotics. Pretreatment with antibiotics significantly decreases the sensitivity of traditional culture methods, making them more likely to produce false-negative results [25]. Antibiotic pretreatment > 12 h before lumbar puncture altered CSF glucose and protein but not CSF WBC count [26]. Antibiotic pretreatment decreases the reliability of CSF cultures, and CSF sterilization has been previously observed after starting antibiotic therapy for *N. meningitidis* within 2 h and *S. pneumoniae* within 4 h [20]. In our study, bacterial pathogens were identified by CSF multiplex panel in 13 patients with negative CSF culture, the majority of whom had been treated with antibiotics (Table 4). We conclude that a multiplex panel is a crucial diagnostic tool allowing detection of pathogen genetic material in pretreated patients. Third, multiplex panel can identify hard-to-culture organisms such as Cryptococcus. None of our patients had cryptococcal meningitis.

Although not assessed in our study, healthcare cost is potentially decreased by multiplex panel. Simultaneous testing of various common pathogens significantly reduces the need for manpower in the lab. In children with viral meningitis, the antibiotic duration is decreased, shortening their stay in the hospital. Time is brain. Early identification of treatable pathogens is expected to decrease morbidity. A randomized prospective study comparing outcomes in patients undergoing or not undergoing CSF multiplex panel is not ethically feasible.

CSF multiplex panel is an essential adjunctive test but has some limitations and is not an all-inclusive library of pathogens. Many viruses such as west nile virus and eastern equine encephalitis virus are not covered by standard CSF multiplex panels. CSF multiplex panel does not cover certain “tropical” pathogens because the panel is designed for the most common etiologies of meningitis in our regions. Pathogens causing subacute and chronic infections, such as syphilis, *Mycobacterium tuberculosis*, *Leptospira* species, most parasites and fungi, just to name a few, are not covered by CSF multiplex panel. Finally, CSF multiplex panel is not recommended to be performed in the CSF from shunt as it does not include Staphylococcus species, coagulase-negative staphylococci, *Cutibacterium acnes*, or Corynebacterium species, which are common causes of shunt infection [9]. Therefore, a negative CSF multiplex panel does not exclude meningitis. We must be mindful that the result of the CSF multiplex panel must be considered alongside the patient’s clinical picture, overall laboratory data, CSF analysis, CSF culture, and imaging studies. Our study shows that the serum CRP level is useful as an adjunct test, significantly higher in bacterial meningitis compared to viral meningitis and this finding is consistent with existing literature [27, 28]. If there is suspicion based on clinical presentation and CSF parameters, further evaluation is needed.

Our study has limitations. It is a single center retrospective study with a small sample size. It assesses only the positive results of CSF multiplex. We are unable to evaluate the sensitivity of CSF multiplex as assessment of false negatives is not performed.

## Conclusions

In summary, this study has shown that the use of CSF multiplex panel helps save children’s brain as it is rapid, specific and highly sensitive. CSF multiplex panel detects the causative pathogen in viral or bacterial meningitis, even when CSF parameters are normal or inadequate. It prevents the use of unnecessary antiviral agents and optimizes the use of specific antiviral agents. It detects bacterial organisms even when Gram stain and culture are negative, especially in pretreated patients. Finally, it rapidly identifies difficult to grow organisms. Overall, the benefits of CSF multiplex panel are multifaceted.

## Supplementary Material

Supplementary Files

This is a list of supplementary files associated with this preprint. Click to download.

- Table6.docx

Table 6

Table 6 is available in the Supplementary Files section.

## Figures and Tables

**Table 1:** Demographic and clinical characteristics of patients who had meningitis and pathogens identified by cerebrospinal fluid polymerase chain reaction multiplex panel^a^

Characteristic	Total	Viral	Bacterial	*p* Value^e^
Total [Count (%)]	79 (100)	58 (73)	21 (27)	
Sex [Count (%)]				0.53
Boys	46 (58)	35 (60)	11 (52)	
Girls	33 (42)	23 (40)	10 (48)	
Age (d)^b^ [Count (%)]				0.39
< 30	33 (42)	25 (43)	8 (38)	
30–90	28 (35)	22 (38)	6 (29)	
> 90	18 (23)	11 (19)	7 (33)	
Presenting symptom^c^ [Count (%)]				
Fever	51 (65)	40 (69)	11 (52)	
Respiratory distress^d^	14 (18)	4 (7)	10 (48)	
Seizure	5 (6)	3 (5)	2 (10)	
Hypothermia	4 (5)	4 (7)	0 (0)	
Lethargy	4 (5)	2 (3)	2 (10)	
Altered mental status	2 (3)	1 (2)	1 (5)	

a N = 79 patients. Data reported as no. of patients (%).

b Mean ± SD: viral, 0.8 ± 1 y; bacterial, 0.5 ± 1 y (difference not significant).

c Total, 80 presenting symptoms because there were 54 presenting symptoms in patients with viral and 26 presenting symptoms in patients with bacterial meningitis. Some patients had more than one of the symptoms mentioned above.

d Respiratory distress: viral, 4 of 58 patients (7%); bacterial, 10 of 21 patients (48%); *P* < .001.

e Both sex and age distributions are statistically similar between the viral and bacterial meningitis groups (p > 0.05 for both).

**Table 2:** Comparison of serum WBC and CRP values in viral and bacterial meningitis

Biomarkers†	Bacterial (n = 20)	Viral (n = 51)	P (2-sample t-test)	Odds ratio (OR)	95% CI For OR	P Value For OR
Serum CRP (mg/dL) ^†^	4.41 (0–24)	3.29 (0–9.7)	< 0.0001	1.28	1.09–1.49	0.002
Serum WBC (cells/mm^3^)^†^	9.36 (4.5–26.37)	11.92 (3.18–31.87)	0.116	1.02	0.92–1.13	0.73

***Abbreviations*** CRP, C-reactive protein; WBC, white blood cell count.

**Table 3:** Cerebrospinal fluid analysis in patients (N = 75) who had meningitis and pathogens identified by cerebrospinal fluid polymerase chain reaction multiplex panel^a^

Variable	Viral (Antibiotic Pretreatment count 5)				Bacterial (Antibiotic Pretreatment count 14)				Comparison of Total Counts	
	< 30 d	30–90 d	> 90 d	Total^b^	< 30 d	30–90d	> 90 d	Total^b^	Odds Ratio (95% CI)	*P*^c^ (Fisher’s test)
No. of patients	22	22	10	54	8	6	7	21		-
Most common No. (%)	Enterovirus 18 (89%)	Enterovirus 14 (64%)	Enterovirus 5 (50%)	-	*Streptococcus agalactiae* 5 (63%)	*Escherichia coli* 3 (50%)	*Haemophilus influenzae* 3 (43%)	-		-
Gram negative	NA	NA	NA	-	2 (25%)	3 (50%)	4 (57%)	9 (43%)		-
Normal CSF protein	19 (86%)	17 (77%)	7 (70%)	43 (80%)	3 (38%)	2 (33%)	2 (29%)	7 (33%)	7.7 (2.4–25.0)	< 0.001
Normal CSF glucose	19 (86%)	19 (86%)	10 (100%)	48 (89%)	6 (75%)	4 (67%)	2 (29%)	12 (57%)	6 (1.8–20.4)	0.01
Normal CSF WBC	14 (64%)	12 (55%)	3 (30%)	29 (54%)	4 (50%)	4 (67%)	1 (14%)	9 (43%)	1.5 (0.6–4.2)	0.46

a N = 75 patients who had CSF data. Data reported as no. of patients or no. of patients (%). In the other 4 patients, CSF glucose level, protein level, and cell count were not performed because of inadequate CSF sample volume, including 3 patients who had viral meningitis caused by cytomegalovirus (1 patient), herpes simplex virus 2 (1 patient), and human herpesvirus 6 (1 patient)and 1 infant aged 4 mo who had bacterial meningitis caused by *E. coli*; 3 of these 4 patients were aged less than 30 days old.

b Statistical comparison between age groups is not performed because of small sample sizes.

c Comparison between total patients with bacterial versus viral meningitis. Viral meningitis is more likely to have normal CSF protein and glucose levels, but CSF WBC counts do not differ significantly.

***Abbreviations:*** CSF, cerebrospinal fluid; NA, not available; WBC, white blood cell.

**Table 4:** Pathogens identified by cerebrospinal fluid polymerase chain reaction multiplex panel and treatment in patients who had meningitis^a^

Pathogen	Total	Age (d)			Treatment duration (d)^b^		
		< 30	30–90	> 90	Acyclovir	Antibiotics^c^	Hospital stay (d)^d^
Virus							
Enterovirus	37 (47)	18 (55)	14 (50)	5 (28)	0.17 (0–3)	2.3 (0–6)	3.48 (1–23)
Human herpesvirus 6	11 (14)	3 (9)	4 (14)	4 (22)	0.43 (0–3)	1.4 (0–3)	4.43 (2–14)
Herpes simplex virus 1 and 2	4 (5)	1 (3)	1 (4)	2 (11)	21 (21)	4 (4)	23 (23)
Human parechovirus	4 (5)	2 (6)	2 (7)	0 (0)	1.25 (0–5)	2 (0–3)	4 (2–6)
Cytomegalovirus^e^	1 (1)	1 (3)	0 (0)	0 (0)	0	2	7
Varicella-zoster virus	1 (1)	0 (0)	1 (4)	0 (0)	14	2	14
Total (virus)	58	25	22	11			
Bacteria							
*Escherichia coli K1*	6 (8)	1 (3)	3 (11)	2 (11)	0 (0)	24.5 (21–27)	23.5 (20–27)
*Streptococcus agalactiae*	6 (8)	5 (15)	1 (4)	0 (0)	0 (0)	22.5 (22–23)	22 (21–23)
*Streptococcus pneumoniae*	5 (6)	1 (3)	2 (7)	2 (11)	0 (0)	9 (9)	27 (27)
*Haemophilus influenzae*	4 (5)	1 (3)	0 (0)	3 (17)	0 (0)	14 (14)	16 (16)
Total (bacterial)	21	8	6	7			
Total	79 (100)	33 (100)	28 (100)	18 (100)			

a N = 79 patients. Data reported as no. of patients (%) or mean (range, minimum to maximum).

b N = 49 patients after excluding patients treated in neonatal and pediatric intensive care units.

c Mean antibiotic treatment duration: viral, 2 d (range, 0–6 d); bacterial, 18 d (range, 9–28 d) (*P* = 0.0015).

d Mean duration of hospital stay: viral, 4 d (1–23 d); bacterial, 22 d (range, 16–27 d) (*P* < 0.0001).

**Table 5:** Cerebrospinal fluid risk in patients who had meningitis and bacterial pathogens identified by cerebrospinal fluid polymerase reaction multiplex panel^a^

CSF pathogen on multiplex panel	Total	Antibiotic pretreatment		No antibiotic pretreatment	
		Growth	No growth	Growth	No growth
*Streptococcus agalactiae*	6	1	2	3	0
*Escherichia coli*	6	0	5	1	0
*Streptococcus pneumoniae*	5	1	2	1	1
*Haemophilus influenzae*	4	0	3	1	0
Total	21	2	12	6	1

a Data reported as no. of patients. N = 21 patients who had bacterial pathogens identified on CSF PCR multiplex panel and had CSF culture results available. There was 1 other patient who had *E. coli* identified by CSF PCR multiplex panel but did not have a CSF culture result available. Antibiotic pretreatment total is 14 patients; No antibiotic treatment total is 7 patients.

Antibiotic pretreatment was strongly associated with reduced CSF culture positivity. Only 14% (2/14) of pretreated patients had positive cultures compared with 86% (6/7) of those without pretreatment. Pretreatment was associated with a markedly lower odds of culture growth (OR 0.03, 95% CI 0.003–0.28, *p* = 0.004, Fisher’s exact test).

***Abbreviations***: CSF, cerebrospinal fluid; PCR, polymerase chain reaction.

## Data Availability

Please contact the corresponding author for any raw data. Kamal Sharma, MD, Ksharma@health.southlabama.edu

## Abbreviations

CMV: cytomegalovirus
CSF: cerebrospinal fluid
h: hours
d: days
y: years
HHV-6: Human herpes virus 6
HSV: Herpes simplex virus
PCR: polymerase chain reaction
VZV: varicella zoster virus
